# Need for post-operative outpatient appointments after discharge following cervical spinal surgery - a narrative review

**DOI:** 10.1186/s12891-020-03624-4

**Published:** 2020-09-15

**Authors:** Karel de Bree, Femke Atsma, Erik J. van Lindert, Gert P. Westert, Ronald M. H. A. Bartels

**Affiliations:** 1 Department of Neurosurgery, Institute for Health Sciences, Radboud University Medical Centre, Postbus 9101, Geert Grooteplein Zuid 10, Nijmegen, 6500 HB The Netherlands; 2 Scientific Center for Quality of Healthcare, Institute for Health Sciences, Radboud University Medical Centre, Postbus 9101, Geert Grooteplein 21, Nijmegen, 6500 HB The Netherlands

**Keywords:** Post-operative, Outpatient, Appointment, Spinal, Surgery, Omitting

## Abstract

**Background:**

In the Netherlands most patients are currently seen in an outpatient clinic after an anterior cervical discectomy, which is an effective neurosurgical procedure with relatively low rate of severe complications. In this back sight, the need for patients returning to the post-operative outpatient clinic could be questioned. The aim of the study is to evaluate whether a post-operative outpatient appointment after anterior cervical discectomy could be replaced by an alternative or be omitted without adversely impacting, or increasing, the value of healthcare and patient satisfaction for this procedure.

**Methods:**

A narrative review was performed to evaluate the quality of care and patient satisfaction for patients with and without a post-operative outpatient appointment after spinal surgery. A literature search of the previous ten years was performed in Pubmed, CENTRAL and EMBASE.

**Results:**

A total of 403 articles were identified. Four studies remained after title and abstract selection by 3 independent reviewers. No papers were selected for further analysis, due to the absence of interventional studies that compared the utility of a post-operative outpatient clinic appointment with an intervention after spinal surgery.

**Conclusions:**

Currently, there is a lack of evidence for the need of a post-operative follow-up after anterior cervical discectomy. Nor is there any literature in favor of omitting these appointments. No determinants which patients benefits from these outpatient appointments could be identified. Potential harmful and beneficial effects of omitting these post-operative follow-ups should be investigated to identify possible determinant for patients who might benefit from a post-operative appointment.

## Background

Anterior cervical discectomy without fusion (ACD), with fusion (ACDF) or arthroplasty (ACDA) is a standard neurosurgical procedure for cervical radiculopathy or cervical stenosis. Success rates are up to 94% for cervical radiculopathy and the procedure has a relatively low rate for serious and permanent complications [1]. Supporting evidence suggests that this procedure can be performed safe and effectively in the outpatient clinic [2]. This reduces hospital admission time, and with anticipation on potential complications in high risk patients, reduces admission related complications [3]. Furthermore, it reduces the patient disease burden and social costs. This illustrates efficient healthcare management and promoting high value healthcare for a neurosurgical procedure resulting in reduction of unnecessary interventions, healthcare costs and potentially increases patient satisfaction and the quality of care [4, 5].

Increasing healthcare costs are an important subject of public debate resulting in a constant pressure to economize these. Cutting healthcare budgets is an ineffective, and even potentially harmful management strategy. Identifying and eliminating drivers of poor medical care is a far more effective strategy to reduce healthcare costs [6, 7]. It is currently common practice for all patients to return to the outpatient clinic after neurosurgical procedures after six to eight weeks. This results from healthcare professionals having adopted occupational habits as a result of their training. It is unknown if these post-operative follow-ups are contributing value of care for patients. Additionally, there is no rationale why these patients should return to the outpatient clinic after six to eight weeks. In lumbar surgery, Bartels et al. already demonstrated that regular, post-operative appointments at the outpatient clinic can be questioned [8]. Post-operative appointments may even be considered an example of overuse of healthcare in the absence of clear scientific evidence of contribution to the value of healthcare [9]. Therefore, alternatives or omitting unnecessary post-operative outpatient appointments might be an elegant way to reduce costs in healthcare and to decrease unnecessary burden to the patient. A more targeted approach may be more effective, in which post-operative visits only will be applied in those patients who really benefit from it or appreciate these follow-ups. However, a prerequisite is that the quality of care and the satisfaction of patients should at least be equal or, even better, increase.

Personal experience in our hospital made us question the benefit of post-operative visits for ACD, ACDF or ACDA. Patient who experience complications visit the emergency department before the follow-up and the follow-up often does not have a medical topic. We hypothesize that the post-operative outpatient appointment after ACD ACDF or ACDA can be omitted or replaced by an alternative consultation without any consequences for the quality of care and improves patients’ satisfaction. The aim of the study is to evaluate the literature comparing the quality of care and patient satisfaction for patients with a post-operative outpatient appointment after spinal surgery to patients without a post-operative outpatient appointment or an alternative consultation. Additionally, we try to identify the patient population or determinants of patients that might benefit from these post-operative appointments.

## Methods

A systematic review was performed, which in search of all literature about the quality of care and post-operative outpatient appointments for spinal surgery in the last ten years. The study focused on all spinal surgical procedures, including ACD, ACDF or ACDA, in order to obtain a complete overview of experiences with follow-up visits, whereas a pilot study limited to cervical spinal procedures only showed no relevant articles could be included. Furthermore, we aimed to establish potential patient related determinants that identify patients who could benefit from a post-operative outpatient appointment after spinal surgery. However, due to the lack of relevant articles this systematic review was converted to a narrative review.

### Search strategy

The initial search was performed in Pubmed using the concept terms from Table 1 combined with AND operator. Additionally, the search was also performed with the concept terms from Table 1 in CENTRAL and EMBASE databases. All duplicates that were encountered from the three databases were subsequently removed. Only study designs that compare a post-operative outpatient clinic appointment study group with one or more intervention(s) were included. All studies irrespective of the type of intervention(s) were included in the search strategy. Additionally, the papers included patient reported outcome measurements, complication rates, patient satisfaction or quality of care as outcome measurement.

**Table 1:** Overview of the four concept terms that were used to identify all relevant literature about the postoperative outpatient appointment following spinal surgery. The concept terms encompass free text words and Mesh terms to overlap as much of the concept term as possible. *The following filters were used: Language: English, Published: In the last 10 years, study type: All studies were excluded, except: Review, meta-analysis, RCT, Retro- and prospective cohort studies*

Concept	Text words	MeSH terms/sub heading
Spinal surgery	Spine*[tiab] OR Spinal*[tiab] OR verteb*[tiab] OR Thoracic Vertebrae[tiab] OR Lumbar Vertebrae[tiab] OR Cervical Vertebrae[tiab] OR Lumbosacral[tiab] OR Intervertebral Disc Degeneration[tiab] OR Degenerative Spine Disease OR ACDF[tiab] OR Anterior Cervical Discectomy*[tiab] OR Anterior Cervical Discectomy and fusion[tiab] OR Discectomy[tiab] OR Interlaminar decompression[tiab] OR Laminectom*[tiab]	“Thoracic Vertebrae”[Mesh] OR “Spine”[Mesh] OR “Lumbosacral Region”[Mesh] OR “Lumbar Vertebrae”[Mesh] OR “Intervertebral Disc Degeneration”[Mesh] OR “Sciatica”[Mesh] OR “Low Back Pain”[Mesh] OR “Diskectomy”[Mesh] OR “Laminectomy”[Mesh]
Post operative appointment	Post operative*[tiab] OR Postoperative*[tiab] OR Post operative management [tiab] OR Postoperative management [tiab] OR Post operative care [tiab] OR Postoperative care[tiab]	“Outpatients”[Mesh] OR “Outpatient Clinics, Hospital”[Mesh] OR “Ambulatory Care”[Mesh] OR Appointments and Schedules[mh] OR Referral and Consultation[mh]
Outpatient	Follow-up consultation [tiab] OR Surgical follow-up [tiab] Ambulatory [tiab] OR Outpatient*[tiab] OR Outpatient*[tiab]	“Outpatients”[Mesh] OR “Outpatient Clinics, Hospital”[Mesh] OR “Ambulatory Care”[Mesh] OR Appointments and Schedules[mh] OR Referral and Consultation[mh]
Quality of care	Quality of care*[tiab] OR Quality of Life*[tiab] OR Complication*[tiab] OR Patient reported outcome measures*[tiab] OR PROM [tiab] OR PROMS [tiab] OR Patiënt satisfaction*[tiab]	“Quality of care”[Mesh] OR “Quality of Health Care”[Mesh] OR “complications”[Subheading]) OR “Postoperative Complications”[Mesh] OR “patient reported outcome measures”[MeSH Terms] OR “patient satisfaction”[MeSH Terms]

In the search strategy four concept terms were used, respectively spinal surgery, post-operative appointment, outpatient and Quality of Care (Table 1), to identify all studies. Search terms of these categories were combined with the AND operator. The search strategy was verified by the medical librarian from the Radboud university medical center. Only studies published in 2017 or the preceding 10 years were included in order to get results that are not outdated.

### Titles and abstract selection

First, a title and abstract selection was performed by three independent observers (3 observers: KdB, EvL and FA) on all studies that were identified with the search strategy. The selected papers were included according to the in-and exclusion criteria (Table 2). Papers were selected for further review in case at least one of the observers selected the abstract. In case one observer selected the abstract and the others did not, the abstract was as yet included in the second step.

**Table 2:** In-and exclusion criteria used for the selection of the titles and abstracts for the three independent observers

Inclusion criteria:	
*1. Study type:* Review, meta-analysis, RCT, Retro-and prospectieve chohortstudies.	
*2. Patients:* Patients who had a spinal surgical intervention above the age of eighteen years.	
*3. Main determinant mentioned in abstract*: Study compares a postoperative outpatient appointment with an intervention (e.g. no appointment, digital appointment, telephone consultation).	
*4. Main outcome measurements mentioned in abstract*: Measurement of quality of care, Complications or Patiënt Reported Outcome Measurement (PROM)	
***Exclusion criteria***	
- Patients with complex spinal surgery, spinal surgery after traumatic injury or patients with	
paraplegia.	
- Patients unable to attend a physical post-operative appointment	

### Paper selection

In the second step full text papers of the selected studies from the abstract selection were screened by the same three observers (KdB, EvL and FA). The same in- and exclusion criteria were used with additional main outcome measurements (Table 3). Studies were included for further review if all three observers selected the full text paper. If only one of the observers selected the paper final consensus between the observers had to be reached about including the study or not. Finally, the reference lists of the selected papers were screened for additional papers for selection by KdB.

**Table 3:** In-and exclusion criteria used for the selection of the papers for the three independent observers

***Inclusion criteria***	
*1. Patients:* Patients who had a spinal surgical intervention above the age of eighteen years.	
2. *Baseline characteristics*: Age, gender, type of spinal surgery, primary or secondary surgical intervention, time of hospitalization.	
3. *Main determinant:* Study compares a physical postoperative outpatient appointment with an intervention (no appointment, digital appointment, telephone consultation).	
4. *Main outcome measurements*: Direct (< 30 days) or late complications related to the surgical intervention and the time interval of occurrence (rate or intensity), time between surgery and complication, measurement of quality of care, time between intervention and postoperative appointment, 6 weeks follow up or longer.	
***Exclusion criteria***	
- Patients with complex spinal surgery, spinal surgery after traumatic injury or patients with paraplegia.	
- Patients unable to attend a physical post-operative appointment	

## Results

**Fig. 1:**
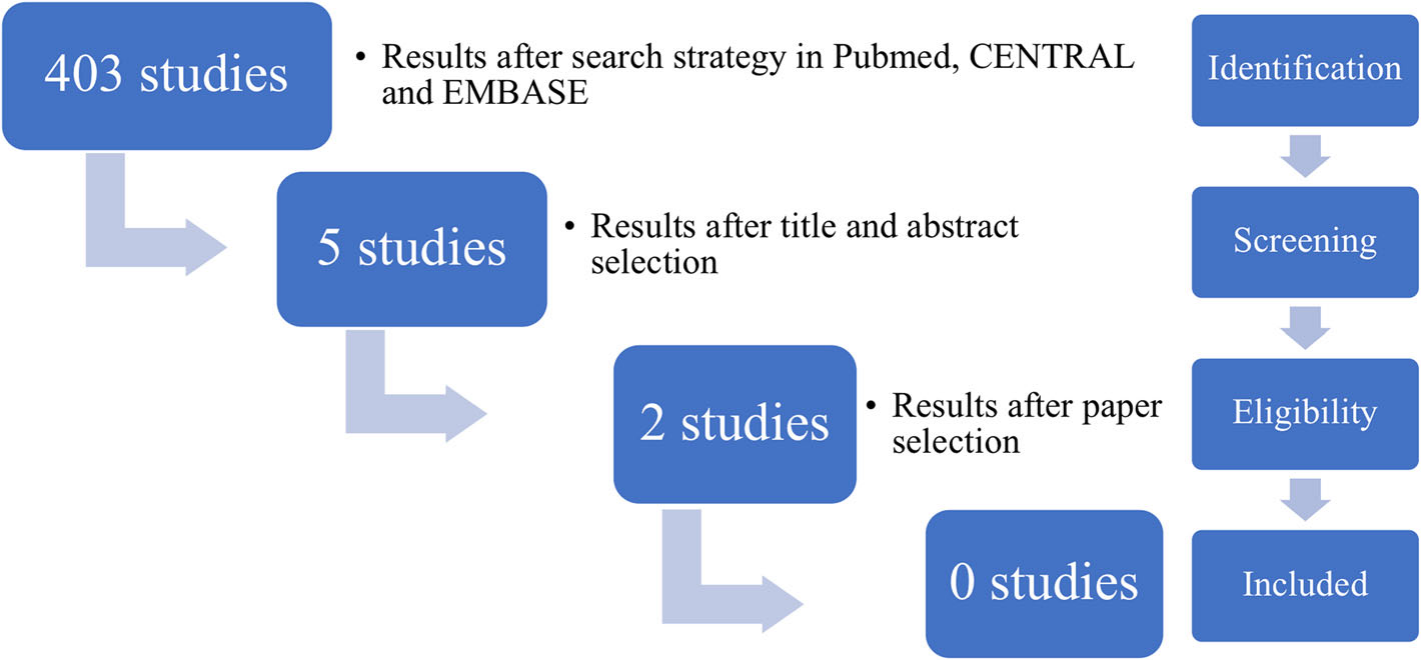
Flowchart of the paper selection process summarized

The literature search yielded 403 studies in Pubmed (Fig. 1). There were no additional papers found in the CENTRAL and EMBASE databases. A total of five studies were selected after screening the title and abstract selection (Table 4). The paper of Debono, et al. was selected by all the independent observers. Consensus was reached about the article of Wohns, et al. for further review, but selected by only one of the observers. The paper of Lied, et al. was selected by one observer and not included for further review after consensus was reached for exclusion (Table 4). The remaining two articles full text papers were printed and subsequently screened according to the in- and exclusion criteria (Fig. 1)(Table 4). Screening of the reference list from the selected papers did not result in extra studies.

**Table 4:** Results of all the selected papers after title and abstract selection. In the column ‘reason’ are arguments noted for in-or exclusion of the papers. In the column consensus is noted if consensus was reached after discussion. KdB: Observer Karel de Bree, EvL: Erik van Lindert, FA: Observer Femke Atsma, Y: Paper selected by observer, N: Paper not selected by observer, I: Inclusion, E: Exclusion

		KdB	EvL	FA	Consensus	Reason	I/E
1	Postoperative monitoring with a mobile application after ambulatory lumbar discectomy: an effective tool for spine surgeons. Debono B, Bousquet P, Sabatier P, Plas JY, Lescure JP, Hamel O. Eur Spine J. 2016 Nov;25(11):3536–3542.	Y		Y	Yes	All inclusion criateria are met.	I
2	Outpatient Surgery for Herniated Cervical Disc and Fusion Is Feasible and Safe: A Consecutive Single-Center Series of 759 Patients. Lied B, Helseth O, Ekseth K, Heskestad B, Helseth E. Neurosurgery. 2016 Aug;63 Suppl 1:164	N		Y	Yes	No results of postoperative outpatient appointment	E
3	Utility of Routine Outpatient Cervical Spine Imaging Following Anterior Cervical Corpectomy and Fusion, Desai A1, Pendharkar AV, Swienckowski JG, Ball PA, Lollis S, Simmons NE, Cureus. 2015 Nov 23;7(11):e387.	Y		N	No	No results of postoperative outpatient appointment	E
4	Safety and cost-effectiveness of outpatient cervical disc arthroplasty. Wohns R. Surg Neurol Int. 2010 Dec 13;1:77.	Y		N	Yes	No results of postoperative outpatient appointment	I
5	Patient satisfaction with outpatient lumbar microsurgical discectomy: a qualitative study. Hersht M, Massicotte EM, Bernstein M. Can J Surg. 2007 Dec;50(6):445–9.	Y		N	No	Qualitative study	E

None of the papers remained after the paper selection, because they did not meet the inclusion criteria. The study of Wohns R. was excluded considering not reporting the outcome after the post-operative outpatient appointment [10]. Additionally, the study of Debono B. et al. did meet all the inclusion criteria in the title and abstract selection [11]. However, there was no comparison between the control group with patients attending the post-operative outpatient appointment and the intervention group that used a mobile application as an intervention. Therefore, this study was also excluded from further analysis.

## Discussion

The literature about post-operative outpatient clinic appointments after spinal surgery was analyzed. No interventional studies were found in the previous ten years that compared the utility of a post-operative outpatient clinic appointment with an intervention after spinal surgery. Apparently, this topic is currently not subject in consideration to create more efficient healthcare logistics and improvement of patient satisfaction in spinal post-operative follow-ups.

### Post-operative follow-up after spinal surgery as targeted care

In our experience the post-operative follow-ups can be more effective, by creating a more targeted care for patients. Unfortunately, any evidence suggesting any benefit or disadvantage for patients is currently not available. Moreover, no subpopulations could be identified to distinguish in which patients the follow-up can be replaced by an alternative, or be omitted, and who benefits from these visits. Usually, patients contact our department when in doubt about the normal post-operative course. Additionally, if adequate information about the post-operative course is pre-operative provided there is not much information left to discuss at the outpatient clinic appointment. Moreover, against the backdrop of value-based healthcare the post-operative follow-up might be considered low value care since it does not contribute to the quality of care, while the post-operative visits require significant healthcare resources. Neurosurgery consultants cannot see new patients if these spots are taken by post-operative patients consuming expensive resources. Additionally these consultations may result in unnecessary diagnostics and may consequently be harmful for the healthcare system which can spend resources only once. It might even be considered as systematic overuse, considering the overlap with published characteristics of overuse procedures [12]. In all, there should be good quality evidence for the need of the post-operative outpatient follow-up after spinal surgery to continue this practice, considering the potential harmful effects on the patients and healthcare system.

### Elimination of poor medical care drivers before considering new technology

Currently it is common using digital applications as a substitute for medical interventions in post-operative follow-ups. The study of Debono B. et al., replaced the standard outpatient post-operative appointment for a mobile application and a telephone call the first post-operative day [11]. They found an increase in patient satisfaction without an increase in complications or decrease in quality of care. Although interventional studies with mobile applications show promising results, the development of applications and studies with multiple study groups are often very expensive and do not necessary increase value of healthcare nor reduce over usage [6]. Less expensive and more elementary alternatives have not been studied for post-operative outpatient follow-ups after spinal surgery. Omitting the post-operative outpatient clinic appointment or replacing it with a telephone consultation by a paramedic might be equal to a mobile application in efficiency and patient satisfaction without extra expenses. These alternatives should be investigated before implementation of the more expensive methods.

### More efficient post-operative follow-ups in other surgical specialties

In other surgical specialties, the discussion about effective logistics in the outpatient clinic follow-up is also subject of debate. Outpatient clinic resources are increasingly demanding and require more effective usage. Bromage et al. studied the urology outpatient follow-up visits and concluded appropriateness and timing of outpatient follow-ups is necessary for more efficient usage of outpatient follow-up recourses [13]. Alternatives for post-operative follow-ups for benign pathology results are being explored to create more time for outpatient clinic visits for new patients. About 50% of the attendances could be avoided for recurrent patients with normal test results, with the potential to optimize outpatient clinic resources [14]. Both Bromage et al. and Gurjar et al. use the earlier mentioned elementary alternatives to create a more efficient outpatient clinic visits. In this saved time, consultants have more time for patients who require more attention, research, education and training their surgical skills for example. The implementation of more effective outpatient follow-ups is an elegant strategy to reduce healthcare resources, thereby creating high value healthcare.

### Increasing value of healthcare with alternatives for post-operative follow-ups

More efficient utilities at the outpatient clinic can not only be beneficial for the quality of care, patient satisfaction and hospital or healthcare costs, but also for patients and possible decreasing the economic burden on society. By implementing (digital) alternatives or skipping the outpatient post-operative appointment after surgery, the patient does not have to travel to the clinic, cancel work or arrange a day-care provider. These non-medical considerations must also be taken in account as a possible positive side effect in an alternative for the post-operative outpatient appointment after spinal surgery. It is also important to consider what makes it worthwhile to attend the outpatient clinic for a patient. Smith and Sanderson concluded that the consultations lasting more than 10 min, receiving advice and having been reassured are important aspects for patients in outpatient consultation [15]. Reassurance, as mentioned by Smith and Sanderson, about the normal post-operative course or doubts about the presence of complications and questions could be an important necessity of the post-operative outpatient consultation for patient satisfaction. However, this study was performed in 1992 when the current communication possibilities were not available. The modern techniques facilitate management of expectations, consultation for minor problems and reassurance. Although skipping the regular outpatient clinic appointment after spinal surgery and its replacement by alternative methods using modern communication technology can be efficient, it is currently unclear if this is in the best interest for the patient satisfaction and value of healthcare.

### Potential harmful effects omitting post-operative follow-ups

Although the fore mentioned arguments favor omitting the outpatient appointment, the disadvantages and potential harmful effects should also be explored. Determinants of patients who might benefit from an outpatient appointment after ACD, ACDA or ACDF should be identified. High risk patients for post-operative complications should be identified and may need a post-operative follow-up. Adequate discharge education of patients about the normal post-operative course and complications enhances the participation in their healthcare management [16]. Additionally, mismatch in expectancy of patient and doctor in different healthcare domains could lead to overrating low value healthcare [6]. Therefore, adequate preoperative information and post-operative discharge education of patients is essential. High risk patients and patients susceptible for misinterpretation of information at discharge might benefit from a post-operative outpatient appointment. Alternatively, the impact on performance of the surgeon by omitting outpatient post-operative appointments should also be explored. There is a difference in the need for post-operative outpatient appointments for trainees and non-spine specialized neurosurgeons compared to experienced spinal surgery consultants for whom this is a routine procedure. The effect of missing feedback or reassurance from patients in the residual symptoms from degenerative disease, the technique used or indication might be essential for non-spine specialized surgeons, especially in training. Besides the patient and surgeon related aspects, there are also differences in society, cultural and level of healthcare to consider. The quality of care in a highly centralized and specialized healthcare system, were omitting might be opportune, can differ greatly between countries or even regions. Lastly, possible problems that could be detected and managed at the standard post-operative consultation, such as wound problems or postoperative pain, could be missed when omitting these visits. The question remains if the visits are necessary for detecting such problems or that alternatives that are suitable can be found. Considerations about omitting or implementing alternatives for postoperative outpatient appointments the aforementioned aspects need to be taken into account.

## Conclusion

In conclusion, any evidence in favor or disfavor for post-operative follow-ups after spinal surgery, and ACD, ACDA or ACDF in particular, related to the quality of healthcare or to patients’ satisfaction does not exist. With the current literature search no determinants could be identified which patients benefits from these outpatient appointments, thereby hindering the possibility to create a more effective post-operative course for patients after spinal surgery. The need for the post-operative outpatient appointment after ACD, ACDA or ACDF can be questioned and should be further explored. Specifically, elementary alternatives for the post-operative appointment after high volume neurosurgical procedures, such as ACD, ACDA or ACDF, should be explored before digital alternatives are introduced.

## Data Availability

Data sharing not applicable as no datasets generated and/or analysed for this study.

## Abbreviations

ACD: Anterior cervical discectomy without fusion
ACDA: Anterior cervical discectomy with arthroplasty
ACDF: Anterior cervical discectomy with fusion
